# *N*-Butyl-4-hydr­oxy-2-methyl-2*H*-1,2-benzothia­zine-3-carboxamide 1,1-dioxide

**DOI:** 10.1107/S160053680801670X

**Published:** 2008-06-07

**Authors:** Matloob Ahmad, Hamid Latif Siddiqui, Saeed Ahmad, Syed Umar Farooq, Masood Parvez

**Affiliations:** a Institute of Chemistry, University of the Punjab, Lahore 54590, Pakistan; b Department of Chemistry, University of Science and Technology, Bannu, Pakistan; c Department of Chemistry, The University of Calgary, 2500 University Drive NW, Calgary, Alberta, Canada T2N 1N4

## Abstract

The title compound, C_14_H_18_N_2_O_4_S, contains hydrogen-bonded dimeric pairs of mol­ecules arranged around inversion centers, forming 14-membered rings with an *R*_2_^2^(14) motif. The structure is stabilized by extensive intra­molecular inter­actions. The thia­zine ring adopts a half-chair conformation, with the S and N atoms displaced by −0.485 (3) and 0.296 (3) Å, respectively, from the plane formed by the remaining atoms of the ring.

## Related literature

For related literature, see: Ahmad, Siddiqui, Ahmad *et al.* (2008 ▶); Ahmad, Siddiqui, Zia-ur-Rehman *et al.* (2008 ▶); Bernstein *et al.* (1994 ▶); Gupta *et al.* (1993 ▶, 2002 ▶); Kojić-Prodić & Rużić-Toroš (1982 ▶); Lombardino (1971 ▶); Lombardino & Wiseman (1972 ▶); Rehman *et al.* (2005 ▶, 2006 ▶); Sianesi *et al.* (1973 ▶); Siddiqui *et al.* (2008 ▶); Zinnes *et al.* (1982 ▶); Drebushchak *et al.* (2006 ▶).
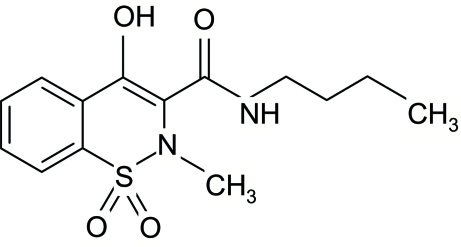


## Experimental

### 

#### Crystal data

C_14_H_18_N_2_O_4_S
*M* *_r_* = 310.36
Monoclinic, 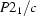

*a* = 10.233 (2) Å
*b* = 14.780 (4) Å
*c* = 10.365 (5) Å
β = 108.79 (2)°
*V* = 1484.1 (9) Å^3^
*Z* = 4
Mo *K*α radiation
μ = 0.23 mm^−1^
*T* = 173 (2) K
0.14 × 0.12 × 0.06 mm

#### Data collection

Nonius KappaCCD diffractometer
Absorption correction: multi-scan (*SORTAV*; Blessing, 1997 ▶) *T*_min_ = 0.968, *T*_max_ = 0.986
12380 measured reflections
3405 independent reflections
2646 reflections with *I* > 2σ(*I*)
*R*_int_ = 0.041

#### Refinement

*R*[*F* ^2^ > 2σ(*F* ^2^)] = 0.039
*wR*(*F* ^2^) = 0.100
*S* = 1.03
3405 reflections
198 parameters
H atoms treated by a mixture of independent and constrained refinement
Δρ_max_ = 0.30 e Å^−3^
Δρ_min_ = −0.43 e Å^−3^

### 

Data collection: *COLLECT* (Hooft, 1998 ▶); cell refinement: *HKL* *DENZO* (Otwinowski & Minor, 1997 ▶); data reduction: *SCALEPACK* (Otwinowski & Minor, 1997 ▶); program(s) used to solve structure: *SHELXS97* (Sheldrick, 2008 ▶); program(s) used to refine structure: *SHELXL97* (Sheldrick, 2008 ▶); molecular graphics: *ORTEP-3 for Windows* (Farrugia, 1997 ▶); software used to prepare material for publication: *SHELXL97*.

## Supplementary Material

Crystal structure: contains datablocks Global, I. DOI: 10.1107/S160053680801670X/bh2176sup1.cif

Structure factors: contains datablocks I. DOI: 10.1107/S160053680801670X/bh2176Isup2.hkl

Additional supplementary materials:  crystallographic information; 3D view; checkCIF report

## Figures and Tables

**Table 1:** Hydrogen-bond geometry (Å, °)

*D*—H⋯*A*	*D*—H	H⋯*A*	*D*⋯*A*	*D*—H⋯*A*
O3—H3O⋯O4	0.88 (2)	1.76 (2)	2.572 (2)	153 (2)
N2—H2N⋯O2^i^	0.87 (2)	2.21 (2)	3.052 (2)	161 (2)
N2—H2N⋯N1	0.87 (2)	2.34 (2)	2.753 (2)	109 (2)
C9—H9*B*⋯O2	0.98	2.49	2.864 (2)	102

Symmetry code: (i) 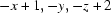.

**Fig. 1.**
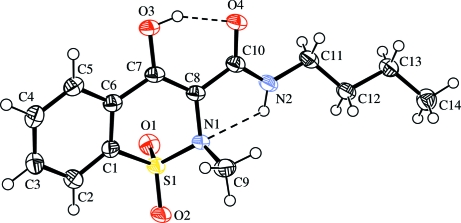
ORTEP-3 (Farrugia, 1997) drawing of (I) with displacement ellipsoids plotted at 50% probability level; intramolecular interactions have been indicated by dashed lines.

**Fig. 2.**
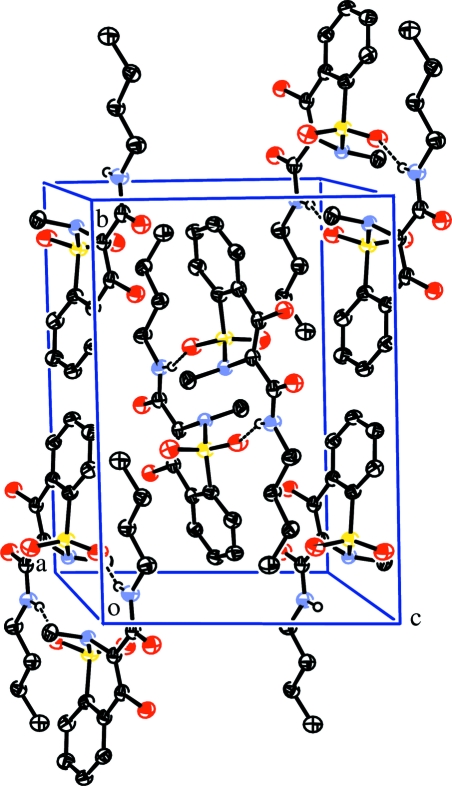
Unit cell packing of (I) showing hydrogen bonds with dashed lines; H atoms not involved in hydrogen bonds have been omitted.

## supplementary crystallographic information

### Comment

Several benzothiazine derivatives like piroxicam, sudoxicam (Lombardino & Wiseman, 1972; Rehman *et al.*, 2005) and isoxicam (Zinnes *et al.*, 1982) have been reported in the literature to be potential anti-inflammatory agents. Some of the derivatives of benzothiazines are found to be analgesic (Gupta *et al.*, 2002), anti-cancer (Gupta *et al.*, 1993) and exhibitors of central nervous system activity (Sianesi *et al.*, 1973). We have reported anti-bacterial activities (Rehman *et al.*, 2006) of a series of 1,2-benzothiazines. In continuation of our work on 1,2-benzothiazines 1,1-dioxides (Ahmad, Siddiqui, Ahmad, Irfan Ashiq & Tizzard, 2008; Ahmad, Siddiqui, Zia-ur-Rehman, Ashiq & Tizzard, 2008), we report in this paper the crystal structure of the title compound, (I), which was patented for Pfizer Inc. (Lombardino, 1971).

The structure of (I), (Fig. 1), contains dimeric pairs of molecules lying about inversion centers resulting from N2—H2N···O2 hydrogen bonds (Fig. 2). The 14-membered rings thus formed represent *R*_2_^2^(14) motif in the graph set notation (Bernstein *et al.*, 1994). Similar hydrogen-bonded dimers have been reported in structures related to the title compound (Siddiqui *et al.*, 2008; Drebushchak *et al.*, 2006; Kojić-Prodić & Rużić-Toroš, 1982). The structure is stabilized by extensive intramolecular interactions (Fig. 1 and Table 1). The thiazine ring in (I) adopts a half-chair conformation with atoms S1 and N1 displaced by -0.485 (3) and 0.296 (3) Å, respectively, from the plane formed by the remaining atoms of the ring.

### Experimental

Methyl-4-hydroxy-2-methyl-2*H*-1,2-benzothiazine-3-carboxylate-1,1-dioxide (1.0 g, 3.72 mmoles) was dissolved in *n*-butyl amine (5 ml) in a test tube. The mixture was placed at room temperature for 7 days. Crystals of (I) suitable for crystallographic analysis were found, which were washed with MeOH.

### Refinement

Though all the H atoms could be found in a difference map, the H atoms bonded to C atoms were included at geometrically idealized positions and refined in riding-model approximation with the following constraints: C—H distances were set to 0.95, 0.98 and 0.99 Å for aryl, methyl and methylene H atoms, respectively, and *U*_iso_(H) = 1.2*U*_eq_(C). H atoms bonded to N2 and O3 were taken from a difference map and were allowed to refine with *U*_iso_ = 1.2 times *U*_eq_ of the parent atom. The final difference map was free of any chemically significant features.

### Crystal data

**Table:**

C_14_H_18_N_2_O_4_S	*F*_000_ = 656
*M**_r_* = 310.36	*D*_x_ = 1.389 Mg m^−^^3^
Monoclinic, *P*2_1_/*c*	Mo *K*α radiation λ = 0.71073 Å
Hall symbol: -P 2ybc	Cell parameters from 12380 reflections
*a* = 10.233 (2) Å	θ = 3.4–27.6º
*b* = 14.780 (4) Å	µ = 0.24 mm^−^^1^
*c* = 10.365 (5) Å	*T* = 173 (2) K
β = 108.79 (2)º	Prism, colorless
*V* = 1484.1 (9) Å^3^	0.14 × 0.12 × 0.06 mm
*Z* = 4	

### Data collection

**Table:**

Nonius KappaCCD diffractometer	3405 independent reflections
Radiation source: fine-focus sealed tube	2646 reflections with *I* > 2σ(*I*)
Monochromator: graphite	*R*_int_ = 0.041
*T* = 173(2) K	θ_max_ = 27.6º
ω and φ scans	θ_min_ = 3.4º
Absorption correction: Multi-scan(SORTAV; Blessing, 1997)	*h* = −13→13
*T*_min_ = 0.968, *T*_max_ = 0.986	*k* = −19→19
12380 measured reflections	*l* = −13→13

### Refinement

**Table:**

Refinement on *F*^2^	Secondary atom site location: difference Fourier map
Least-squares matrix: full	Hydrogen site location: inferred from neighbouring sites
*R*[*F*^2^ > 2σ(*F*^2^)] = 0.039	H atoms treated by a mixture of independent and constrained refinement
*wR*(*F*^2^) = 0.100	*w* = 1/[σ^2^(*F*_o_^2^) + (0.045*P*)^2^ + 0.606*P*] where *P* = (*F*_o_^2^ + 2*F*_c_^2^)/3
*S* = 1.03	(Δ/σ)_max_ = 0.001
3405 reflections	Δρ_max_ = 0.30 e Å^−^^3^
198 parameters	Δρ_min_ = −0.43 e Å^−^^3^
Primary atom site location: structure-invariant direct methods	Extinction correction: none

### Fractional atomic coordinates and isotropic or equivalent isotropic displacement parameters (Å^2^)

**Table:**

	*x*	*y*	*z*	*U*_iso_*/*U*_eq_	
S1	0.53051 (4)	0.13868 (3)	0.96872 (5)	0.03033 (13)	
O1	0.45310 (12)	0.12905 (9)	0.82760 (14)	0.0384 (3)	
O2	0.46724 (13)	0.11547 (9)	1.06916 (15)	0.0400 (3)	
O3	0.86081 (13)	0.22923 (9)	0.83438 (13)	0.0336 (3)	
H3O	0.899 (2)	0.1799 (16)	0.816 (2)	0.050*	
O4	0.92527 (12)	0.06200 (9)	0.82242 (13)	0.0356 (3)	
N1	0.67274 (13)	0.07980 (9)	0.99855 (14)	0.0265 (3)	
N2	0.78434 (15)	−0.04424 (10)	0.86553 (16)	0.0327 (3)	
H2N	0.714 (2)	−0.0525 (14)	0.894 (2)	0.039*	
C1	0.59359 (17)	0.24980 (11)	0.99677 (17)	0.0283 (4)	
C2	0.53309 (18)	0.31325 (12)	1.05849 (19)	0.0342 (4)	
H2	0.4587	0.2967	1.0897	0.041*	
C3	0.58309 (19)	0.40116 (12)	1.07376 (19)	0.0359 (4)	
H3	0.5430	0.4455	1.1159	0.043*	
C4	0.69144 (19)	0.42436 (12)	1.02763 (18)	0.0349 (4)	
H4	0.7235	0.4851	1.0363	0.042*	
C5	0.75381 (17)	0.36033 (12)	0.96906 (18)	0.0309 (4)	
H5	0.8289	0.3772	0.9392	0.037*	
C6	0.70664 (17)	0.27103 (11)	0.95385 (17)	0.0276 (4)	
C7	0.77716 (16)	0.19970 (12)	0.90287 (17)	0.0272 (4)	
C8	0.76003 (16)	0.11021 (12)	0.92283 (17)	0.0269 (4)	
C9	0.74832 (19)	0.05636 (13)	1.14249 (18)	0.0355 (4)	
H9A	0.8206	0.0121	1.1457	0.043*	
H9B	0.6839	0.0305	1.1848	0.043*	
H9C	0.7905	0.1110	1.1921	0.043*	
C10	0.82934 (17)	0.04054 (12)	0.86719 (17)	0.0288 (4)	
C11	0.8470 (2)	−0.12047 (12)	0.8176 (2)	0.0385 (4)	
H11A	0.9478	−0.1107	0.8430	0.046*	
H11B	0.8091	−0.1238	0.7170	0.046*	
C12	0.82004 (18)	−0.20820 (12)	0.8775 (2)	0.0335 (4)	
H12A	0.7192	−0.2189	0.8486	0.040*	
H12B	0.8537	−0.2034	0.9781	0.040*	
C13	0.88952 (19)	−0.28900 (12)	0.83487 (19)	0.0339 (4)	
H13A	0.8496	−0.2975	0.7351	0.041*	
H13B	0.9892	−0.2761	0.8562	0.041*	
C14	0.8724 (2)	−0.37560 (13)	0.9058 (2)	0.0405 (5)	
H14A	0.9237	−0.4245	0.8799	0.049*	
H14B	0.7743	−0.3916	0.8787	0.049*	
H14C	0.9079	−0.3668	1.0048	0.049*	

### Atomic displacement parameters (Å^2^)

**Table:**

	*U*^11^	*U*^22^	*U*^33^	*U*^12^	*U*^13^	*U*^23^
S1	0.0252 (2)	0.0286 (2)	0.0417 (3)	−0.00243 (16)	0.01704 (18)	−0.00165 (18)
O1	0.0263 (6)	0.0414 (8)	0.0450 (8)	−0.0039 (5)	0.0078 (5)	−0.0038 (6)
O2	0.0400 (7)	0.0340 (7)	0.0597 (9)	−0.0031 (5)	0.0352 (7)	−0.0005 (6)
O3	0.0347 (7)	0.0328 (7)	0.0411 (7)	−0.0041 (5)	0.0232 (6)	0.0015 (6)
O4	0.0316 (6)	0.0379 (7)	0.0455 (8)	−0.0004 (5)	0.0239 (6)	0.0008 (6)
N1	0.0267 (7)	0.0273 (7)	0.0306 (8)	−0.0003 (5)	0.0160 (6)	0.0018 (6)
N2	0.0306 (8)	0.0311 (8)	0.0433 (9)	−0.0001 (6)	0.0215 (7)	−0.0031 (7)
C1	0.0269 (8)	0.0267 (8)	0.0330 (9)	0.0003 (6)	0.0121 (7)	0.0023 (7)
C2	0.0317 (9)	0.0342 (10)	0.0404 (10)	0.0042 (7)	0.0167 (8)	0.0028 (8)
C3	0.0400 (10)	0.0296 (9)	0.0393 (10)	0.0073 (8)	0.0142 (8)	0.0019 (8)
C4	0.0407 (10)	0.0269 (9)	0.0353 (10)	−0.0007 (7)	0.0097 (8)	0.0024 (7)
C5	0.0306 (9)	0.0309 (9)	0.0315 (9)	−0.0027 (7)	0.0104 (7)	0.0040 (7)
C6	0.0255 (8)	0.0307 (9)	0.0267 (8)	−0.0011 (7)	0.0087 (7)	0.0019 (7)
C7	0.0229 (8)	0.0338 (9)	0.0264 (8)	−0.0028 (6)	0.0098 (7)	0.0014 (7)
C8	0.0234 (8)	0.0318 (9)	0.0282 (9)	−0.0018 (6)	0.0122 (7)	0.0008 (7)
C9	0.0385 (10)	0.0390 (10)	0.0331 (10)	−0.0019 (8)	0.0171 (8)	0.0021 (8)
C10	0.0245 (8)	0.0345 (9)	0.0290 (9)	0.0006 (7)	0.0107 (7)	0.0003 (7)
C11	0.0432 (10)	0.0326 (10)	0.0502 (12)	0.0037 (8)	0.0294 (9)	−0.0028 (8)
C12	0.0324 (9)	0.0340 (10)	0.0398 (10)	0.0013 (7)	0.0194 (8)	−0.0026 (8)
C13	0.0329 (9)	0.0348 (10)	0.0367 (10)	0.0015 (7)	0.0152 (8)	−0.0036 (8)
C14	0.0365 (10)	0.0356 (10)	0.0486 (12)	−0.0003 (8)	0.0128 (9)	−0.0003 (9)

### Geometric parameters (Å, °)

**Table:**

S1—O1	1.4286 (15)	C5—C6	1.397 (2)
S1—O2	1.4335 (14)	C5—H5	0.9500
S1—N1	1.6375 (14)	C6—C7	1.469 (2)
S1—C1	1.7543 (18)	C7—C8	1.359 (2)
O3—C7	1.349 (2)	C8—C10	1.470 (2)
O3—H3O	0.88 (2)	C9—H9A	0.9800
O4—C10	1.254 (2)	C9—H9B	0.9800
N1—C8	1.438 (2)	C9—H9C	0.9800
N1—C9	1.484 (2)	C11—C12	1.501 (3)
N2—C10	1.333 (2)	C11—H11A	0.9900
N2—C11	1.460 (2)	C11—H11B	0.9900
N2—H2N	0.87 (2)	C12—C13	1.526 (2)
C1—C2	1.388 (2)	C12—H12A	0.9900
C1—C6	1.402 (2)	C12—H12B	0.9900
C2—C3	1.387 (3)	C13—C14	1.514 (3)
C2—H2	0.9500	C13—H13A	0.9900
C3—C4	1.385 (3)	C13—H13B	0.9900
C3—H3	0.9500	C14—H14A	0.9800
C4—C5	1.386 (3)	C14—H14B	0.9800
C4—H4	0.9500	C14—H14C	0.9800
			
O1—S1—O2	119.25 (9)	C7—C8—C10	121.34 (15)
O1—S1—N1	107.84 (8)	N1—C8—C10	117.28 (14)
O2—S1—N1	108.51 (8)	N1—C9—H9A	109.5
O1—S1—C1	108.42 (8)	N1—C9—H9B	109.5
O2—S1—C1	109.38 (8)	H9A—C9—H9B	109.5
N1—S1—C1	102.07 (8)	N1—C9—H9C	109.5
C7—O3—H3O	104.4 (15)	H9A—C9—H9C	109.5
C8—N1—C9	114.06 (13)	H9B—C9—H9C	109.5
C8—N1—S1	113.67 (11)	O4—C10—N2	122.76 (16)
C9—N1—S1	117.24 (11)	O4—C10—C8	120.15 (15)
C10—N2—C11	122.83 (15)	N2—C10—C8	117.08 (15)
C10—N2—H2N	116.6 (14)	N2—C11—C12	111.55 (15)
C11—N2—H2N	120.6 (14)	N2—C11—H11A	109.3
C2—C1—C6	122.08 (16)	C12—C11—H11A	109.3
C2—C1—S1	120.92 (13)	N2—C11—H11B	109.3
C6—C1—S1	117.00 (13)	C12—C11—H11B	109.3
C3—C2—C1	118.85 (16)	H11A—C11—H11B	108.0
C3—C2—H2	120.6	C11—C12—C13	113.08 (14)
C1—C2—H2	120.6	C11—C12—H12A	109.0
C4—C3—C2	119.98 (17)	C13—C12—H12A	109.0
C4—C3—H3	120.0	C11—C12—H12B	109.0
C2—C3—H3	120.0	C13—C12—H12B	109.0
C3—C4—C5	121.00 (17)	H12A—C12—H12B	107.8
C3—C4—H4	119.5	C14—C13—C12	112.53 (15)
C5—C4—H4	119.5	C14—C13—H13A	109.1
C4—C5—C6	120.23 (16)	C12—C13—H13A	109.1
C4—C5—H5	119.9	C14—C13—H13B	109.1
C6—C5—H5	119.9	C12—C13—H13B	109.1
C5—C6—C1	117.78 (16)	H13A—C13—H13B	107.8
C5—C6—C7	121.80 (15)	C13—C14—H14A	109.5
C1—C6—C7	120.31 (15)	C13—C14—H14B	109.5
O3—C7—C8	122.01 (15)	H14A—C14—H14B	109.5
O3—C7—C6	115.20 (15)	C13—C14—H14C	109.5
C8—C7—C6	122.79 (15)	H14A—C14—H14C	109.5
C7—C8—N1	121.38 (14)	H14B—C14—H14C	109.5
			
O1—S1—N1—C8	61.67 (13)	S1—C1—C6—C7	−6.9 (2)
O2—S1—N1—C8	−167.86 (11)	C5—C6—C7—O3	−19.3 (2)
C1—S1—N1—C8	−52.42 (13)	C1—C6—C7—O3	164.56 (15)
O1—S1—N1—C9	−161.75 (12)	C5—C6—C7—C8	160.65 (16)
O2—S1—N1—C9	−31.28 (14)	C1—C6—C7—C8	−15.5 (2)
C1—S1—N1—C9	84.16 (13)	O3—C7—C8—N1	178.67 (14)
O1—S1—C1—C2	103.81 (15)	C6—C7—C8—N1	−1.3 (2)
O2—S1—C1—C2	−27.73 (17)	O3—C7—C8—C10	−1.7 (3)
N1—S1—C1—C2	−142.53 (15)	C6—C7—C8—C10	178.33 (15)
O1—S1—C1—C6	−76.03 (15)	C9—N1—C8—C7	−98.65 (19)
O2—S1—C1—C6	152.43 (13)	S1—N1—C8—C7	39.3 (2)
N1—S1—C1—C6	37.63 (15)	C9—N1—C8—C10	81.71 (18)
C6—C1—C2—C3	2.3 (3)	S1—N1—C8—C10	−140.30 (13)
S1—C1—C2—C3	−177.53 (14)	C11—N2—C10—O4	3.1 (3)
C1—C2—C3—C4	0.1 (3)	C11—N2—C10—C8	−177.77 (16)
C2—C3—C4—C5	−1.7 (3)	C7—C8—C10—O4	13.4 (3)
C3—C4—C5—C6	0.9 (3)	N1—C8—C10—O4	−166.94 (15)
C4—C5—C6—C1	1.4 (2)	C7—C8—C10—N2	−165.76 (16)
C4—C5—C6—C7	−174.82 (16)	N1—C8—C10—N2	13.9 (2)
C2—C1—C6—C5	−3.0 (3)	C10—N2—C11—C12	156.43 (17)
S1—C1—C6—C5	176.82 (13)	N2—C11—C12—C13	−177.35 (16)
C2—C1—C6—C7	173.25 (16)	C11—C12—C13—C14	174.83 (17)

### Hydrogen-bond geometry (Å, °)

**Table:**

*D*—H···*A*	*D*—H	H···*A*	*D*···*A*	*D*—H···*A*
O3—H3O···O4	0.88 (2)	1.76 (2)	2.572 (2)	153 (2)
N2—H2N···O2^i^	0.87 (2)	2.21 (2)	3.052 (2)	161 (2)
N2—H2N···N1	0.87 (2)	2.34 (2)	2.753 (2)	109 (2)
C9—H9B···O2	0.98	2.49	2.864 (2)	102

Symmetry codes: (i) −*x*+1, −*y*, −*z*+2.
